# Electroacupuncture for post-stroke urinary incontinence: a systematic review and meta-analysis with trial sequential analysis

**DOI:** 10.3389/fneur.2023.1282580

**Published:** 2023-11-15

**Authors:** Zefei Jiang, Na Zhi, Guang Liu, Xiaoxiang Sun, Xi Chen, Dandan Ma, Mingming Guo, Siying Wang, Hong Zhang

**Affiliations:** Acupuncture and Tuina School, Chengdu University of Traditional Chinese Medicine, Chengdu, Sichuan, China

**Keywords:** electroacupuncture, post-stroke urinary incontinence, meta-analysis, systematic review, trial sequential analysis

## Abstract

**Background:**

The evidence for the effectiveness of electroacupuncture (EA) for post-stroke urinary incontinence (PSUI) patients remains unclear. Therefore, the purpose of this systematic review and meta-analysis was to assess the efficacy of EA for PSUI.

**Methods and analysis:**

Eight English and Chinese databases were searched from their inception until 1 August 2023 to collect randomized controlled trials (RCTs) that investigated the effect of EA on PSUI. Two reviewers independently selected studies that met the eligibility criteria, extracted the necessary data, and assessed the risk of bias for included studies using Cochrane Handbook version 5.1.0. Meta-analysis was performed using Review Manager software (version 5.4.1). Publication bias detection was conducted using STATA (version 16.0). Sequential analysis was performed using TSA 0.9.5.10 Beta. The Grading of Recommendations Assessment, Development, and Evaluation System (GRADE) was used for assessing the certainty of evidence.

**Results:**

We included 15 RCTs involving a total of 1,414 patients. The narrative analysis revealed that compared with sham EA, genuine EA exhibited greater efficacy in reducing occurrences of 24-h urinary incontinence while also enhancing maximum cystometric capacity (MCC). Moreover, this effect remained significant even during the 3-month follow-up period. Fourteen studies were encompassed within the quantitative analysis. In contrast to active interventions, EA did not yield an improvement in the responder rate (RR 1.53, 95% CI 0.61 to 3.80, *p* = 0.36). When compared with basic treatments, the combination of EA with them led to a reduction in 24-h urinary incontinence occurrences (MD −0.56, 95% CI −0.60 to −0.52, *p* < 0.00001), an improvement in MCC (MD 43.23, 95% CI 28.86 to 57.60, *p* < 0.00001), and a decrease in residual urine volume (RUV; MD −19.99, 95% CI −29.75 to −10.23, *p* < 0.0001). However, it did not lead to an increase in the responder rate (RR 1.39, 95% CI 0.88 to 2.20, *p* = 0.16). In comparison to basic treatments combined with active interventions, the amalgamation of EA and them led to an increase in the responder rate (RR 1.24, 95% CI 1.14 to 1.35, *p* < 0.00001), a reduction in 24-h urinary incontinence occurrences (MD −2.90, 95% CI −5.26 to −0.55, *p* = 0.02), a decrease in International Consultation on Incontinence Questionnaire-Short Form scores, and an improvement in both MCC (MD 42.11, 95% CI 23.26 to 60.96, *p* < 0.0001) and RUV (MD 42.11, 95% CI 23.26 to 60.96, *p* < 0.0001). Furthermore, all reported adverse effects associated with EA were mild. The trial sequential analysis suggested that a sufficient sample size was available to yield results. However, the level of evidence was predominantly assessed as low or very low.

**Conclusion:**

Electroacupuncture improved post-stroke urinary incontinence with no serious adverse effects. Caution is warranted due to methodological issues, and more high-quality studies are required to confirm its efficacy and safety.

**Systematic Review Registration:**https://www.crd.york.ac.uk/prospero/display_record.php?ID=CRD42023449599, Identifier CRD42023449599.

## 1 Introduction

Stroke is the second leading cause of disability and death worldwide (1, 2), with an estimated 13.7 million new stroke cases in 2016 (1, 2). In China, it has taken the lead as the primary cause of death and disability. Statistics for 2020 indicate that approximately 17.8 million Chinese adults encountered a stroke, leading to 2.3 million deaths (3). Stroke survivors may suffer from various complications, such as aphasia, hemiplegia, swallowing difficulties, urinary incontinence (UI), and fecal incontinence. Urinary incontinence is characterized by urinary frequency, urgency, and uncontrolled urine flow from the urethra. A study from Germany reported that the prevalence of urinary incontinence in male stroke survivors was 22%, while in female stroke survivors, it was 34% (4). The pathological mechanisms of post-stroke urinary incontinence (PSUI) are not fully understood, and the primary cause of urinary dysfunction in stroke patients is damage to the anterior cingulate gyrus, its descending pathways, and the basal ganglia (5). In addition, the parietal lobe and white matter lesions are also involved in this process (6). PSUI carries adverse effects, contributing to reduced quality of life (7), increased risk of infections (8), skin problems, psychological distress, sleep disturbances, and challenges for caregivers. Furthermore, in comparison to post-stroke patients without UI, those suffering from UI have higher rates of hospitalization and increased disability (9). Therefore, the management of PSUI requires attention and emphasis.

The management of PSUI involves a comprehensive approach, including behavioral interventions, physical therapy, pharmacotherapy, and, if necessary, surgical options. Nonetheless, most of the evidence quality remains low (10). Bladder training and pelvic floor exercises require sustained commitment from patients over the long term. Anticholinergic drugs are commonly employed for the treatment of PSUI although they carry the potential for side effects such as dry mouth and facial flushing due to their anticholinergic properties. These medications can impede bladder muscle contractility, potentially resulting in urinary retention and exacerbating constipation (11) (*Management of Urinary Incontinence in Older Adults in Rehabilitation Care Settings*). Surgical intervention carries not only a significant cost but also the potential for a range of complications (12). Therefore, the treatment methods for PSUI need to continue to be developed.

The history of acupuncture traces back thousands of years to ancient China, and electroacupuncture has evolved as a modern variation of traditional acupuncture. It involves the application of low electrical currents through acupuncture needles, enhancing the therapeutic effects of the treatment. Over the years, some high-quality clinical studies have found that electroacupuncture (EA) treatment has therapeutic effects on certain diseases, including chronic pain (13), constipation (14), insomnia (15), and knee osteoarthritis (13, 14, 16). In 2017, a clinical research paper was published in *JAMA* (17), indicating that EA treatment for stress urinary incontinence among women resulted in a notable reduction in urinary leakage compared to sham EA. In addition to enhanced local brain region functional activities and functional connections (18, 19), EA also has anti-inflammatory effects (20) and can mitigate brain damage and the activation of glial cells caused by acute ischemic stroke (21). It also decreases the accumulation of damaged mitochondria (22). As of now, several systematic reviews have demonstrated the effectiveness of EA in the treatment of female urinary incontinence (23–25). Similarly, EA is widely used in China for PSUI and has been supported by several clinical studies (26–29). However, there is currently no systematic review/meta-analysis of EA for PSUI. Therefore, we conducted this study to determine the clinical efficacy and safety of EA in treating PSUI and performed a trial sequential analysis (TSA) to ascertain whether the sample size was sufficient.

## 2 Methods and analysis

This study was conducted in accordance with the PRISMA 2020 statement: an updated guideline for reporting systematic reviews (16) and was registered on the PROSPERO website.^1^

### 2.1 Literature research

We have performed a literature search, covering the time span from the inception of the databases until 1 August 2023. The language was limited to both Chinese and English. For the Chinese database, we searched the following databases: China National Knowledge Infrastructure (CNKI), Wanfang, Cqvip (VIP), and China Biomedical Literature Service System (CBM). As for the English database, we searched through PubMed, Embase, CENTRAL, and Web of Science. The keywords included “stroke,” “cerebrovascular accident,” “cerebral infarction,” “urinary incontinence,” “urinary incontinence, urge,” “urinary incontinence, stress,” “diurnal enuresis,” “involuntary urination,” “leaking of urine,” “electroacupuncture,” “acupuncture,” “electric acupuncture,” “randomized controlled trials” “randomized,” “randomly,” and “RCT,.” The detailed retrieval process can be found in Supplementary material.

1. *Inclusion criteria:* We included studies that met the following criteria.
2. *Types of studies:* Only randomized controlled trials (RCTs) were eligible for inclusion, while quasi-RCTs, cohort studies, cluster RCTs, and case reports were excluded.
3. *Types of participants:* The patients included in this study are adults who met the recognized diagnostic criteria for PSUI, irrespective of stroke type, type of urinary incontinence, gender, disease duration, or race.
4. *Types of intervention group:* We included EA as an intervention for the treatment of PSUI in our systematic review. The experimental group included EA and EA combined with basic treatments (referring to the treatment of the primary stroke etiology, such as blood pressure management, anticoagulation, antiplatelet therapy, and symptomatic treatment) or EA added to basic stroke treatment along with active treatments (referring to bladder function training, intermittent catheterization, and medication therapy conducted for UI). There are no restrictions on acupoint selections, EA waveform, current intensity, or treatment duration.
5. *Types of control group:* The control group consists of basic treatments for stroke and/or active treatments for urinary incontinence, as well as sham acupuncture and a waiting list, among other interventions.
6. *Types of outcome measures:* The primary outcome measure was the responder rate; the responder rate was defined as the proportion of participants who demonstrate a positive response within the total number of participants in the group. The secondary outcome was the improvement in 24 h urinary incontinence frequency, International Consultation on Incontinence Questionnaire-Short Form (ICIQ-SF), maximum cystometric capacity (MCC), residual urine volume (RUV), and incidence of adverse reactions.

### 2.2 Study selection and data extraction

First, two reviewers independently conducted literature searches using the same retrieval scheme and imported the search results into Noteexpress (version 3.2.0). Duplicate publications were removed. Then, two reviewers individually read the titles and abstracts to exclude obviously irrelevant studies, such as reviews, case reports, and animal experiments and then cross-checked their exclusions. Next, full-text articles were downloaded, and two reviewers thoroughly read the articles based on the inclusion and exclusion criteria. In case of any disagreements, a third reviewer was consulted to reach a consensus on the final inclusion of articles.

The final selected articles were subjected to data extraction by two reviewers using a standardized form. The extracted data included information on study design, authors, publication year, baseline characteristics (such as age, gender, stroke type, type of urinary incontinence, and duration of the condition), intervention measures, outcomes, and other relevant details.

### 2.3 Risk of bias (quality) assessment

According to Cochrane Handbook version 5.1.0 (30), the risk of bias assessment was conducted for the included studies, which includes the following six domains: random sequence generation; allocation concealment; blinding of participants and personnel; completeness of outcome data; selective reporting; and other sources of bias. Each criterion was evaluated as “low risk,” “high risk,” or “unclear risk” of bias.

### 2.4 Statistical analysis

The meta-analysis was conducted using RevMan software (version 5.4, the Nordic Cochrane Centre, Copenhagen, Denmark). For dichotomous data (e.g., responder rate), the effect size was expressed as relative risk (RR) with 95% confidence intervals (95% CI). For continuous data (e.g., MCC), the effect size was expressed as mean difference (MD) with 95% CI. A *p*-value of <0.05 is considered statistically significant. The chi-square test and I^2^ statistics were employed to assess the statistical heterogeneity among the included studies. When the studies showed a low homogeneity (*p* ≥ 0.1, I^2^ ≤ 50%), a fixed-effects model was used. However, in cases where significant heterogeneity was present among the studies (*p* < 0.1, I^2^ > 50%), subgroup analysis or sensitivity analysis was conducted based on the characteristics of the data to explore the sources of heterogeneity. If the source of heterogeneity remained unclear, a random-effects model was used for the analysis. In the event that it is not possible to pool the outcome data for meta-analysis, a narrative analysis was presented instead. The analysis for publication bias was performed using STATA software (version 16.0, Stata Corp LP, United States).

### 2.5 Trial sequential analysis

Traditional meta-analyses lack attention to statistical power, and when the number of included trials or sample size is small, approximately 25% of traditional meta-analyses may produce erroneous false-positive conclusions due to the influence of random error (31). TSA overcomes the limitations of traditional meta-analysis and enables the estimation of the required sample size for a meta-analysis and provides a stopping criterion for clinical trials (32). For the TSA, the determination of significance boundaries was performed utilizing the O’Brien-Fleming alpha-spending method. We used a statistical significance level of 5%, a power of 80%, and a reduction in the risk ratio of 25% was considered clinically significant. The required information size (RIS) can be obtained by TSA, which refers to the number of cases required to achieve statistical significance in a meta-analysis. TSA sets a clinical trial-stopping criterion by estimating RIS, reached when the cumulative meta-analysis cases hit the predefined RIS value, allowing trial termination. TSA 0.9.5.10 Beta (Copenhagen Trial Unit, Denmark, www.ctu.dk/tsa) was used for trail sequential analysis.

### 2.6 Quality of evidence

The GRADE (Grading of Recommendations, Assessment, Development, and Evaluation) evaluation tool (33) was used by two reviewers to rank the quality of evidence for the outcomes in terms of the following five dimensions: risk of bias, inconsistency, indirectness, imprecision, and publication bias. The quality of evidence is ranked as either high, moderate, low, or very low. A third reviewer resolved disagreements for consensus.

## 3 Results

### 3.1 Search results

**Figure 1:**
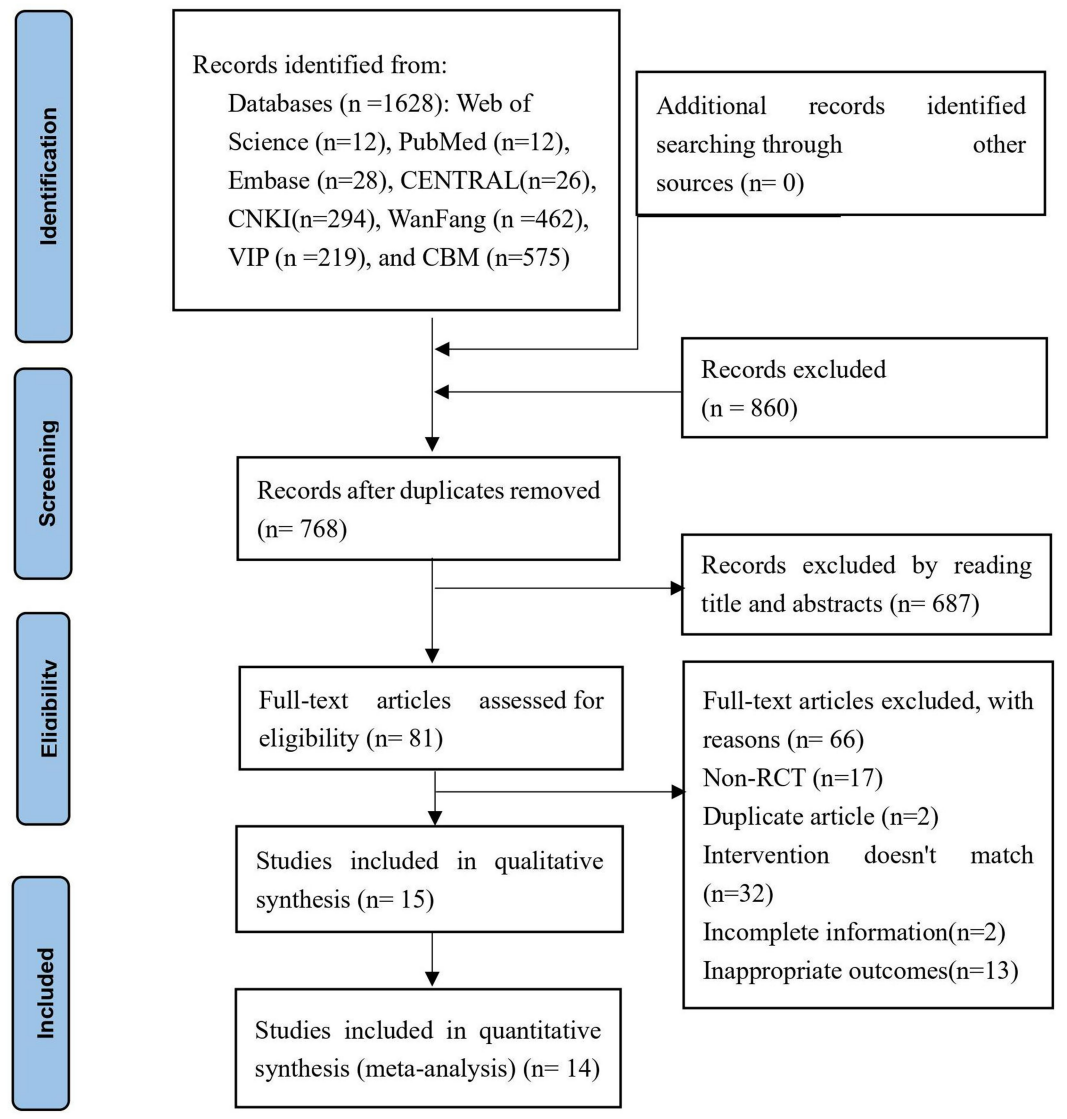
PRISMA flow diagram of the study selection process.

A total of 1,628 studies were identified in the search across eight databases. Among them, 860 duplicates were identified and subsequently removed, leaving 768 studies for further screening. After carefully reviewing the titles and abstracts, 687 studies were excluded based on predetermined criteria, resulting in 81 studies that met the preliminary selection criteria. Subsequently, the full texts of these 81 studies were obtained and thoroughly read. In total, 64 studies were excluded for various reasons, as depicted in Figure 1. Ultimately, 15 studies (26, 34–47) were considered suitable for inclusion in the qualitative synthesis, while 14 studies (34–47) were selected for the quantitative synthesis, as presented in Figure 1.

### 3.2 Studies characteristics

The characteristics of the included studies are presented in Table 1. A total of 15 studies involving 1,414 patients were identified (773 in the experimental group and 641 in the control group). All the studies were conducted in China, with 14 of them published in Chinese (34–47) and one in English (26). Two studies (35, 40) were multicenter randomized controlled trials. All the studies included in this analysis were two-arm studies. The average age of the patients ranged from 37 to 68. In terms of stroke types, with the exception of two studies (one encompassing patients with intracerebral hemorrhage) (34) and one with ischemic stroke (39), all other studies included patients with both stroke and ischemic stroke. In terms of UI types, three studies involved patients with urgency UI (35, 36, 44), while the remaining studies did not report the type of UI. The treatment duration ranged from 4 weeks to 12 weeks. Six studies (35, 39–41, 45, 46) reported adverse reactions.

**Table 1:** Characteristics of included studies.

References	Types of stroke	Type of urinary incontinence	Sample (T/C)	Age		Course of disease (d)		Interventions		Duration	Outcome	Safety
				T	C	T	C	T	C			
Liu and Du (46)	Mixed	NR	102/51	67	68	443.21 ± 912.144	433.49 ± 819.534	EA	AT	40 days	①	R
Song et al. (40)	Mixed	NR	136/68	55 ± 7	54 ± 7	31.2 ± 22.3	29.2 ± 24.2	EA	AT	4 weeks	①	R
Liu et al. (26)	Mixed	NR	35/36	39 ± 12	37 ± 11	-	-	EA	Sham EA	24 days	②⑤	NR
Wang et al. (35)	Mixed	Urge urinary incontinence	80/80	63.96 ± 8.85	63.57 ± 7.03	2.75 ± 0.24 (m)	2.70 ± 0.37 (m)	EA + BT	BT	2 weeks	⑤	R
Wang et al. (38)	Mixed	NR	35/35	-	-	-	-	EA + BT	BT	20 days	①②③	NR
Chu et al. (42)	Mixed	NR	56/55	66.36 ± 9.32	64.58 ± 8.63	35.14 ± 25.56	35.49 ± 23.82	EA + BT	BT	4 weeks	①	NR
Zhou et al. (43)	Mixed	NR	46/36	59.3 ± 15.2	60.1 ± 14.7	-	-	EA + BT	BT	35 days	②③	NR
He (45)	Mixed	NR	30/30	-	-	-	-	EA + BT	BT	1 month	①	NR
Liu et al. (36)	Mixed	Urge urinary incontinence	20/20	54.90 ± 8.44	57.10 ± 12.17	11.85 ± 5.06 (w)	11.10 ± 5.19 (w)	EA + BT	BT	4 weeks	②	NR
Wu and Yang (37)	Mixed	NR	42/42	64.12 ± 9.24	62.84 ± 9.05	20.62 ± 3.25	21.48 ± 2.82	EA + BT +A T	BT + AT	4 weeks	①②③⑤	NR
Nie et al. (34)	Hemorrhagic	NR	21/21	52.95 + 8.30	55.24 ± 11.44	5.10 ± 1.14	5.14 ± 1.15	EA + BT + AT	BT + AT	12 weeks	①③④**⑤**	NR
Liu (47)	Mixed	NR	30/30	67.52 ± 6.14	67.10 ± 7.34	3.7 ± 0.6	3.8 ± 0.1	EA + BT + AT	BT + AT	2 weeks	①②③	NR
Zhao and Hao (39)	Ischemic	NR	72/72	61.21 ± 5.44	60.45 ± 5.06	10.21 ± 1.23	11.02 ± 1.31	EA + BT + AT	BT + AT	3 months	①②	R
Zhang et al. (41)	Mixed	NR	50/47	-	-	-	-	EA + BT +A T	BT + AT	2 weeks	①②③	NR
Chen (44)	Mixed	Urge urinary incontinence	18/18	66.27	67.81	124.4	174.69	EA + BT	BT + AT	4 weeks	①	R

T, test group; C, control group; EA, electroacupuncture; BT, basic treatments; AT, active treatments; Mixed, ischemic and hemorrhagic; R, Report; NR, Not report; d, day; w, week; m, month; ①, Responder rate; ②, Maximum Cystometric Capacity; ③, Residual urine volume; ④, ICIQ-SF; ⑤, 24-h urinary incontinence frequency.

### 3.3 Risk of bias

**Figure 2:**
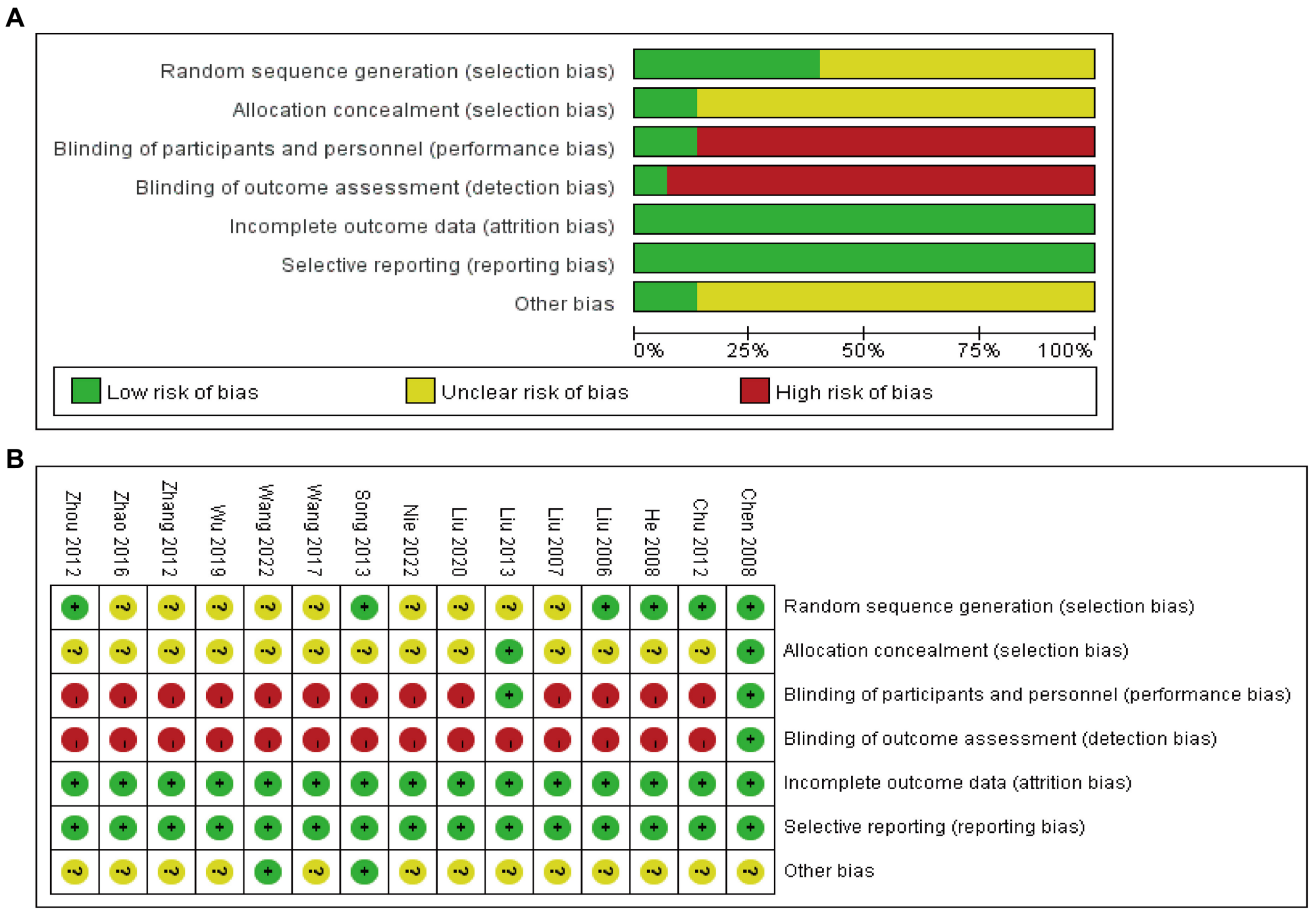
Risk of bias of RCTs: risk of bias graph **(A)** and risk of bias summary **(B)**.

In the generation of random sequences, nine studies (34–41, 46) mentioned the term “random” but did not specify the randomization method, classified as “unclear risk.” The remaining studies were all considered low risk (three studies utilized drawing lots (42, 45, 47), two studies (40, 43) used a random number table, and one study (44) employed computer software for randomization). Regarding allocation concealment, two studies used sealed envelopes (one study with opaque sealed envelopes was considered low risk (44), while the other study (42) did not provide specific details, categorized as “unclear risk”). Another study (26) successfully implemented allocation concealment through a concealment questionnaire, and the remaining studies did not mention allocation concealment. As for blinding of participants and personnel, one study was double-blinded (26), one study (44) involved blinded assessors and data analysts, and the remaining studies did not mention blinding. All studies had dropout rates below 20%, and no selective reporting of study results was found. Among the other sources of bias, two studies (35, 41) were categorized as low risk and involved multicenter randomized controlled clinical trials. The detailed results are shown in Figures 2A,B.

### 3.4 Acupuncture protocols included trials

As depicted in Table 2, all included studies provided details about their choice of acupoints. Among them, the three most frequently utilized acupoints were Ciliao (*n* = 7), Zhongji (*n* = 7), Huiyang (*n* = 6), Guanyuan (*n* = 6), Zhongliao (*n* = 5), Baihui (*n* = 5), and Sanyinjiao (*n* = 5). Except for four studies that did not offer this information, the remaining studies employed acupoints on both sides of the body. Regarding needle stimulation, all studies, except for five studies (34, 39, 42, 46, 47), reported actively seeking the Deqi response. A variety of waveforms were used in the EA parameters, with the continuous waveform being the most frequently employed. Among the four studies (26, 35, 44, 46), the treatment duration was 20 min, while eight studies (34, 36–38, 42, 43, 45, 47) a 30-min duration was utilized; however, the exact treatment duration was not reported in the remaining studies. The majority of the studies implemented a once-daily treatment frequency.

**Table 2:** Electroacupuncture intervention protocols of included randomized controlled trials.

References	Acupoints	Unilateral or bilateral	Deqi	Electroacupuncture parameters	Minutes of each time	Dose or frequency	Duration
Chen (44)	Ciliao (BL32), Zhongliao (BL33), Huiyang (BL35)	Bilateral	Deqi	Sparse-dense wave	20 min	Once a day, 5 times a week.	4 weeks
				Frequency: 20 Hz			
Chu et al. (42)	Sishencong (EX-HN1), Shenshu (UB 23), and Huiyang (BL35)	NR	NR	Sparse wave	30 min	Once a day, 6 times a week.	4 weeks
He (45)	Lieque (LU 7), Sanyinjiao (SP6), Taixi (KI 3), Taichongpoint (LR 3), Zhongji (RN3), and Guanyuan (RN4)	Bilateral	Deqi	Sparse-dense wave	30 min	Once a day	1month
Liu (47)	Shangliao (BL31), Ciliao (BL32), Zhongliao (BL33), Xialiao (BL34), and Huiyang (BL35)	Bilateral	NR	Continuous wave	30 min	Once a day, 6 times a week.	2 weeks
				Frequency: 30~40 Hz			
Liu and Du (46)	Ciliao (BL32), Zhongliao (BL33), and Huiyang (BL35)	Bilateral	NR	Continuous wave	20 min	Once a day, 5 times a week.	40 days
				Frequency: 20 Hz			
Liu et al. (26)	Shangliao (BL31), Ciliao (BL32), Zhongliao (BL33), Xialiao (BL34) and Huiyang (BL35)	Bilateral	Deqi	Continuous wave	20 min	Electroacupuncture was performed daily for 10 consecutive days.	24 days
				Frequency: 30–40Hz			
Liu et al. (36)	Zhongji (RN3), Guanyuan (RN4)	NR	Deqi	Continuous wave	30 min	Once a day, 5 times a week.	4 weeks
				Frequency: 15 Hz			
Nie et al. (34)	Ciliao (BL32)	Bilateral	NR	Dense wave	30 min	Once a day	12 weeks
Song et al. (40)	Qugu (RN2), Zhongji (RN3), Shuidao (ST28), Qihai (RN6), Baihui (GV20), Ezhongxian (MS1), Sishencong (EX-HN1), Zusanli (ST36), and Sanyinjiao (SP6)	Bilateral	Deqi	Sparse-dense wave	NR	5 times a week	4 weeks
				Frequency: 1~10Hz			
				Electrical current: 2~5mA			
Wang et al. (38)	Baihui (GV20), Sishencong (EX-HN1), Shenshu (UB 23), and Huiyang (BL35)	NR	Deqi	Sishencon: Sparse wave	30 min	Once a day	20 days
				Frequency: 2Hz			
				Electrical current: 2 mA			
				Shenshu, Huiyang (BL35):			
				Dense wave			
				Frequency: 100 Hz			
				Electrical current: 2mA			
Wang et al. (35)	Guanyuan (RN4), Zhongji (RN3), and Dahe (KI12)	Bilateral	Deqi	Sparse-dense wave	20 min	Once a day, 5 times a week.	2 weeks
				Frequency: 4/20 Hz			
Wu and Yang (37)	Shangliao (BL31), Ciliao (BL32), Zhongliao (BL33), and Xialiao (BL34)	Bilateral	Deqi	Continuous wave	30 min	Once a day	4 weeks
				Frequency: 10 Hz			
Zhang et al. (41)	Baihui (GV20), Zhongji (RN3), Guanyuan (RN4), Qihai (RN6), Diji (SP 8), Sanyinjiao (SP6), Shenshu (UB 23), and Pangguangshu (BL 28)	NR	Deqi	NR	NR	Once a day, 6 times a week.	2 weeks
Zhao and Hao (39)	Baihui (GV20), Guanyuan (RN4), Zhongji (RN3), Sanyinjiao (SP6), Zusanli (ST36), and Taixi (KI 3)	Bilateral	NR	Electrical current: 0–50 mA	NR	3 times a week	3 months
				Frequency: 15–85 Hz			
				Pulse width: 200–500 μs			
Zhou et al. (43)	Group 1: Baihui (GV20), Sishencong (EX-HN1), Guanyuan (RN4), Zhongji (RN3), and Shuidao (ST28)	NR	Deqi	Continuous wave	30 min	Once a day, 10 consecutive sessions make up one course of treatment, with a 3-day interval between courses.	35 days
	Group 2: Shenshu (UB 23), Pangguangshu (BL 28), Ciliao (BL 32), Yinlingquan (SP 9), Sanyinjiao (SP6), and Taixi (KI 3)			Frequency: 2~15 Hz			

NR, Not report. Deqi: a sensation of numbness and distension at the needling site. Unilateral or bilateral: if acupoints are distributed bilaterally. The abbreviations in the acupoints indicate the unique codes of each acupoint.

### 3.5 Responder rate

**Figure 3:**
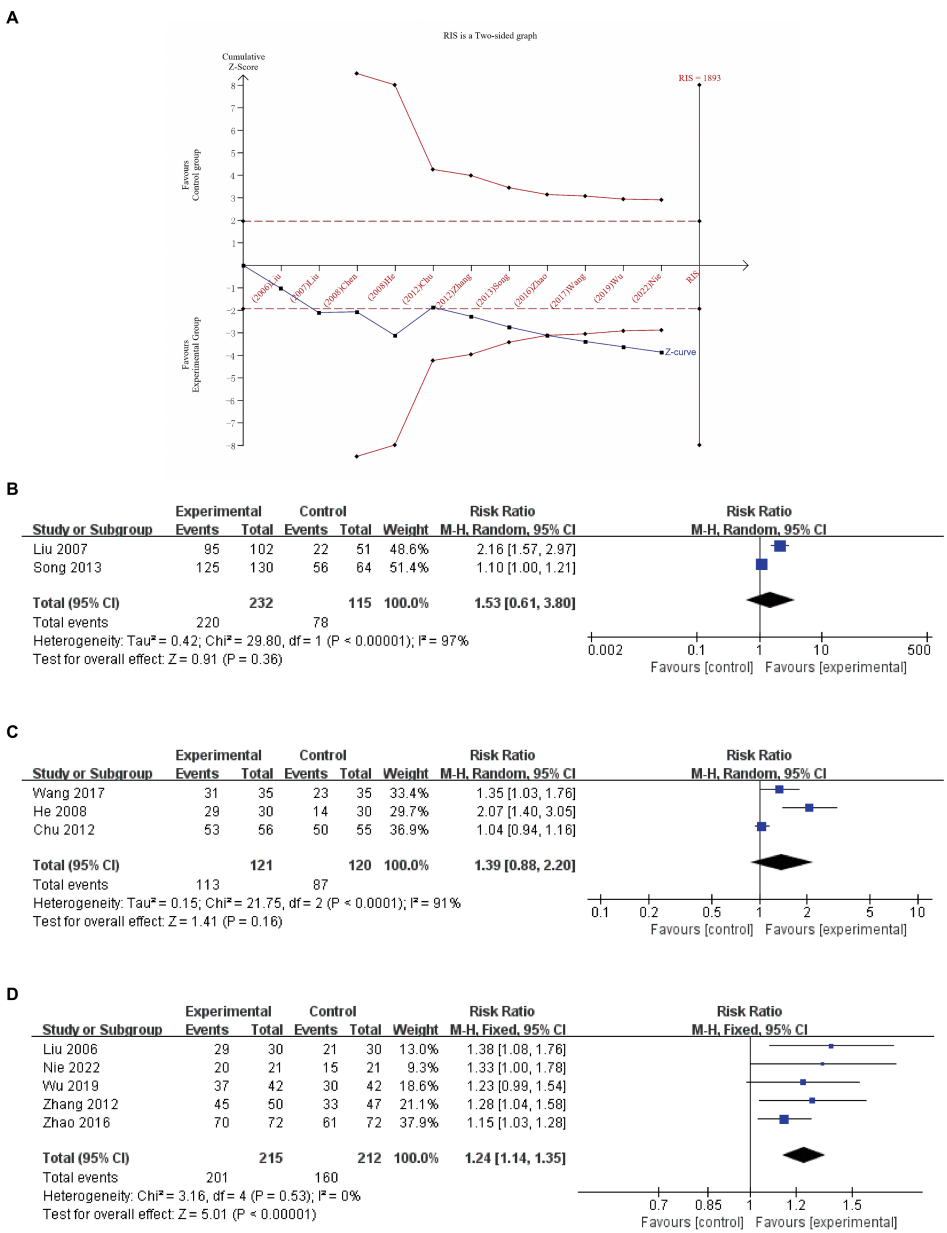
Trial sequential analysis (RIS is a two-sided graph) for responder rate **(A)**; a meta-analysis of responder rate of EA vs. active treatments **(B)**; EA plus basic treatments vs. basic treatments **(C)**; and EA plus basic treatments and active treatments vs. basic treatments plus active treatments **(D)**.

Eleven studies (*n* = 1,051, I^2^ = 82%) (34, 37–42, 44–47) reported the responder rate as an outcome. A random-effects model was employed. The pooled results indicated a significantly higher responder rate in the experimental group compared to the control group (RR 1.32, 95% CI 1.15 to 1.52, *p* = 0.0001, I^2^ = 82%). Converting to a fixed-effects model for analysis and systematically omitting one of the trials did not reverse the results, indicating robustness in the findings. As illustrated in Figure 3A, the TSA analysis demonstrated that the cumulative Z-curve intersected the traditional threshold (indicated by a dashed line) and surpassed the TSA threshold. This suggests that a positive conclusion had been reached before crossing the required information size (RIS) boundary (RIS = 1,893). These findings indicate that acupuncture contributes to an increased treatment response rate in patients with PSUI. Considering the different intervention measures, we performed a subgroup analysis as follows.

#### 3.5.1 EA vs. active treatments

Two studies (*n* = 347, I^2^ = 97%) (40, 46) were pooled to compare EA and active treatments after treatment. Given the substantial heterogeneity, a random-effects model was employed. The results indicated that there was no significant difference in improving the responder rate after treatment (RR 1.53, 95% CI 0.61 to 3.80, *p* = 0.36, Figure 3B).

#### 3.5.2 EA plus basic treatments vs. basic treatments

Three studies (*n* = 241) (38, 42, 45) were pooled to compare the efficacy of EA in addition to basic treatments vs. basic treatments alone. Due to the observed substantial heterogeneity (I^2^ = 91%), we employed a random-effects model for the analysis. The results indicated no significant difference in improving the responder rate after treatment between the two groups (RR 1.39, 95% CI 0.88 to 2.20, *p* = 0.16; Figure 3C).

#### 3.5.3 EA plus basic treatments and active treatments vs. basic treatments plus active treatments

A total of five studies (*n* = 427) (34, 37, 39, 41, 47) compared the combined effects of EA with basic treatments and active treatments vs. the combined effects of basic treatments and active treatments. Given the low heterogeneity observed (I^2^ = 0%), a fixed-effects model was used for the analysis The results of the meta-analysis showed a greater improvement in the experimental group compared to the control group (RR 1.24, 95% CI 1.14 to 1.35, *p* < 0.00001; Figure 3D).

#### 3.5.4 EA plus basic treatments vs. basic treatments plus active treatments

One study (44) compared the efficacy of EA plus basic treatments with basic treatments plus active treatments. The results indicated no statistically significant difference (*p* = 0.06) between the two groups in terms of responder rate.

### 3.6 24-h urinary incontinence frequency

#### 3.6.1 EA vs. sham EA

One study (26) compared EA and sham EA, and the results showed that EA led to a lower incidence of 24-h urinary incontinence frequency compared to sham EA both at the end of treatment and during the 3-month follow-up period (*p* < 0.005).

#### 3.6.2 EA plus basic treatments vs. basic treatments

One study (35) compared EA plus basic treatment and basic treatment. The results showed EA plus basic treatment could decrease the 24-h urinary incontinence frequency (MD −0.56, 95% CI -0.60 to −0.52, *p* < 0.00001).

#### 3.6.3 EA plus basic treatments plus active treatments vs. basic treatments plus active treatments

**Figure 4:**
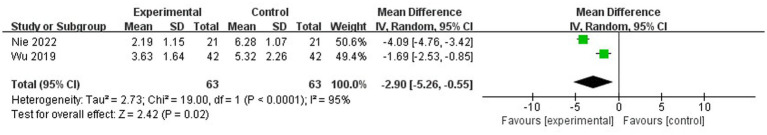
A meta-analysis of 24-h urinary incontinence frequency of EA plus basic treatments plus active treatments vs. basic treatments plus active treatments.

Two studies (*n* = 126) (34, 37) compared the combined effects of EA with basic treatments and active treatments vs. the combined effects of basic treatments and active treatments. Given the high heterogeneity observed (I^2^ = 95%), a random-effects model was used for the analysis. The results indicated that the experimental group performed better than the control group (MD −2.90, 95% CI −5.26 to −0.55, *p* = 0.02, Figure 4).

### 3.7 ICIQ-SF

One study (34) compared EA plus basic treatment with active treatments alone, and basic treatments combined with active treatments, and the result showed that EA leads to more reduction (*p* = 0.0004).

### 3.8 MCC

**Figure 5:**
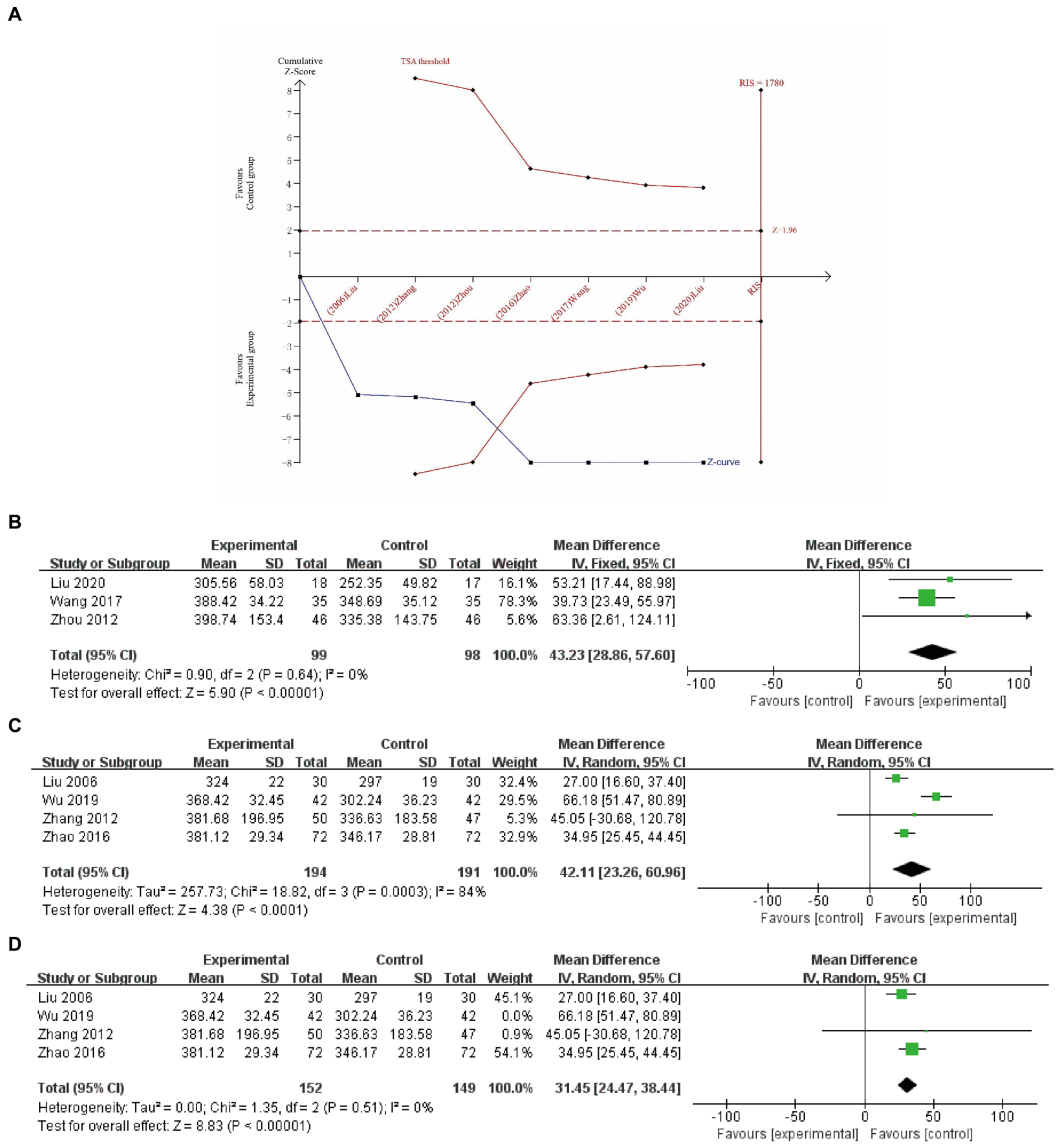
Trial sequential analysis (RIS is a two-sided graph) for MCC **(A)**; a meta-analysis of MCC of EA plus basic treatments vs. basic treatments **(B)**; a meta-analysis of MCC of EA plus basic treatments plus active treatments vs. basic treatments plus active treatments **(C)**; and a study has been excluded **(D)**.

A total of eight studies (26, 36–39, 41, 43, 47) included MCC as an outcome measure. Unfortunately, data from one study (26) could not be extracted, leaving seven studies eligible for inclusion in the meta-analysis. The findings indicated that the experimental group exhibited superior improvements in MCC compared to the control group (MD 38.71, 95% CI 32.93 to 44.49, *p* < 0.00001); however, significant heterogeneity was observed (I^2^ = 70%). Upon exclusion of the study by Wu et al. (37), heterogeneity decreased (MD 33.70, 95% CI 27.42 to 39.98, *p* < 0.00001, I^2^ = 0%). As depicted in Figure 5A, the TSA analysis demonstrated that although the cumulative Z-curve did not cross the RIS boundary, it surpassed the TSA threshold. This indicates that the experimental group exhibited superior improvement in MCC compared to the control group, and a further increase in sample size is unnecessary.

Considering the distinct intervention measures, we performed a subgroup analysis as follows.

#### 3.8.1 EA vs. sham EA

Liu et al. (26) suggested that EA treatment was superior to sham EA in increasing the MCC and still superior to sham EA at the 3-month follow-up.

#### 3.8.2 EA plus basic treatments vs. basic treatments

Three studies (*n* = 197) (36, 38, 43) were conducted to compare the effects of EA in addition to basic treatments vs. basic treatments alone. With low heterogeneity among these studies (I^2^ = 0%), a random-effects model was employed for the analysis. The results demonstrated that the inclusion of EA plus basic treatments significantly improved the MCC (MD 43.23, 95% CI 28.86 to 57.60, *p* < 0.00001, Figure 5B) when compared to basic treatments alone.

#### 3.8.3 EA plus basic treatments plus active treatments vs. basic treatments plus active treatments

A total of four studies (*n* = 385) (37, 39, 41, 47) compared the combined effects of EA with basic treatments and active treatments to the combined effects of basic treatments and active treatments. Given the observed high heterogeneity (I^2^ = 84%), a random-effects model was utilized for the analysis. The results indicated that the experimental group exhibited superior improvements in MCC (MD 42.11, 95% CI 23.26 to 60.96, *p* < 0.0001, refer to Figure 5C). Sensitivity analysis revealed that upon exclusion of the study by Wu et al. (37), heterogeneity decreased substantially (I^2^ = 0%), while a statistically significant difference between the two groups persisted (MD 31.45, 95% CI 24.47 to 38.44, *p* < 0.00001, Figure 5D).

### 3.9 RUV

In total, six studies (34, 37–39, 41, 43) reported the RUV outcome. Due to the observed significant heterogeneity, a random-effects model was utilized for the analysis. The meta-analysis demonstrated that the experimental group exhibited superiority over the control group in reducing RUV (MD −19.66, 95% CI −30.24 to −9.07, *p* = 0.0003).

Considering the distinct intervention measures, we conducted a subgroup analysis as outlined below.

#### 3.9.1 EA plus basic treatments vs. basic treatments

**Figure 6:**
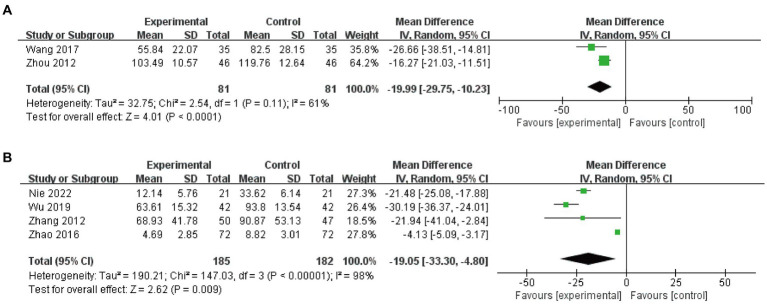
Meta-analysis and RUV of EA plus basic treatments vs. basic treatments **(A)** and EA plus basic treatments plus active treatments vs. basic treatments plus active treatments **(B)**.

Two studies (*n* = 162) (38, 43) were pooled to compare EA plus basic treatments to basic treatments, and a random-effects model was used for the heterogeneity (I^2^ = 61%). The results showed that EA, when used in conjunction with basic treatments, was more effective in reducing RUV compared to basic treatments alone (MD −19.99, 95% CI −29.75 to −10.23, *p* < 0.0001, Figure 6A).

#### 3.9.2 EA plus basic treatments plus active treatments vs. basic treatments plus active treatments

Four studies (*n* = 367, I^2^ = 98%) (34, 37, 39, 41) were included in the analysis, and a random-effects model was used. The results demonstrated that EA significantly improved RUV compared to the control group (MD −19.05, 95% CI -33.30 to −4.80, *p* = 0.009, Figure 6B).

### 3.10 Adverse reactions

A total of five (35, 39, 40, 44, 46) studies reported adverse reaction indicators, among which one study (46) did not report any adverse reactions related to EA. The remaining studies reported minor symptoms such as subcutaneous ecchymosis and mild pain during needle insertion, which could self-resolve within a few hours to 1 week.

### 3.11 Quality of evidence

We utilized the GRADE framework to evaluate the quality of outcome evidence. Our evaluation results (Table 3) revealed the presence of two outcomes supported by moderate-quality evidence, seven outcomes supported by low-quality evidence, and two outcomes supported by very low-quality evidence. Notably, none of the outcomes were substantiated by evidence of high quality. The primary factors contributing to the downgrade in evidence quality are related to experimental design, heterogeneity, and limited sample sizes.

**Table 3:** Quality of evidence included randomized controlled trials (RCTs) by Grading of Recommendations Assessment, Development, and Evaluation (GRADE).

Outcomes	Included RCTs (patients)	Relative effect (95% CI)	Quality assessment								Quality of evidence
			Risk of bias	Inconsistency	Indirectness	Imprecision	Publication bias	Large effect	Dose response	All plausible confounding	
**Responder rate**											
EA vs. AT	2(347)	RR 1.53 (0.61, 3.80)	Serious①	Serious②	Not serious	Not serious	Undetected	Undetected	Undetected	Undetected	⊕⊕○○
											Low
EA plus BT vs. BT	3(241)	RR 1.39 (0.88, 2.20)	Serious①	Serious②	Not serious	Not serious	Undetected	Undetected	Undetected	Undetected	⊕⊕○○
											Low
EA plus BT plus AT vs. BT plus AT	5(427)	RR 1.24 (1.14, 1.35)	Serious①	Not serious	Not serious	Not serious	Undetected	Undetected	Undetected	Undetected	⊕⊕⊕○
											Moderate
EA plus BT vs. BT plus AT	1(36)	RR 15 (0.92, 244.51)	Not serious	Undetected	Not serious	Very serious③	Undetected	Undetected	Undetected	Undetected	⊕○○○
											Low
**24-hour urinary incontinence frequency**											
EA plus BT vs. BT	1(155)	MD −0.56 (−0.60, −0.52)	Serious①	Undetected	Not serious	Serious③	Undetected	Undetected	Undetected	Undetected	⊕⊕○○
											Low
EA plus BT plus AT vs. BT plus AT	2(126)	MD −2.90 (−5.26, −0.55)	Serious①	Serious②	Not serious	Serious③	Undetected	Undetected	Undetected	Undetected	⊕○○○
											Very low
ICIQ-SF	1(42)	MD −1.76 (−2.74, −0.78)	Serious①	Undetected	Not serious	Serious③	Undetected	Undetected	Undetected	Undetected	⊕⊕○○
											Low
**MCC**											
EA plus BT vs. BT	3(197)	MD 43.23 (28.86, 57.60)	Serious①	Not serious	Not serious	Not serious	Undetected	Undetected	Undetected	Undetected	⊕⊕⊕○
											Moderate
EA plus BT plus AT vs. BT plus AT	4(385)	MD 42.11 (23.26, 60.96)	Serious①	Serious②	Not serious	Not serious	Undetected	Undetected	Undetected	Undetected	⊕⊕○○
											Low
**RUV**											
EA plus BT vs. BT	2(162)	MD −19.99 (−29.75, −10.23)	Serious①	Serious②	Not serious	Serious③	Undetected	Undetected	Undetected	Undetected	⊕○○○
											Very low
EA plus BT plus AT vs. BT plus AT	4(367)	MD −19.05 (−33.30, −4.80)	Serious①	Serious②	Not serious	Not serious	Undetected	Undetected	Undetected	Undetected	⊕⊕○○
											Low

EA, electroacupuncture; BT, basic treatment; AT, active treatment; MCC, maximum cystometric capacity; RUV, residual urine volume; Moderate, Moderate quality; Very low, very low quality; Low, low quality; MD, weighted mean difference; RR, risk ratio; CI, confidence interval; ① Most information is from the moderate risk studies, and there are major limitations; ② Significant heterogeneity (I^2^ > 50%); ③ The confidence interval was wide or the sample size was small.

### 3.12 Reporting bias

**Figure 7:**
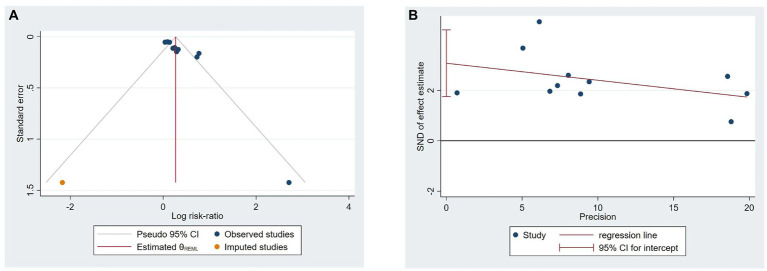
Funnel plots **(A)** and Egger’s test **(B)** of included studies on responder rate.

Due to limitations in reporting the outcomes included in the study, funnel plots and Egger’s tests were only conducted for response rate. The results indicate (Figures 7A,B) that there is publication bias in studies with responder rate as the outcome, as evidenced by Egger’s test with a *p*-value of <0.05.

## 4 Discussion

### 4.1 Summary of main results

We included 15 RCTs with 1,414 subjects and explored the effects and safety of EA as monotherapy or adjunctive therapy. In comparison to sham EA, EA proved to be more effective in reducing occurrences of urinary incontinence and improving MCC. In contrast, when compared with active treatments, EA did not exhibit an improvement in the responder rate. However, when combined with basic treatments, EA demonstrated a capacity to decrease urinary incontinence occurrences and improve MCC and RUV, albeit without a concurrent increase in the responder rate. Furthermore, in comparison to basic treatments combined with active treatments, the integration of EA with them exhibited an elevated responder rate, reduced urinary incontinence occurrences, lowered ICIQ-SF scores, and improved MCC and RUV. Only a few EA-related adverse events were reported. In summary, EA therapy offers certain advantages, especially when used in combination with other treatments, due to its non-pharmacological nature, potential for individualized treatment, and minimal adverse effects, all of which contribute to enhanced treatment efficacy. TSA analysis indicated a sufficient sample size for producing reliable results.

### 4.2 Compared with previous reviews

Currently, there is no existing systematic review or meta-analysis focusing on the utilization of EA for PSUI. Nonetheless, a meta-analysis regarding PSUI has indicated that acupuncture and EA, serving as supplementary and alternative therapies, could potentially enhance the number of participants who maintain continence post-treatment (10, 48). This finding presents some variance in comparison to our study. In our investigation, EA displayed significant differences in improving the responder rate only when synergistically combined with basic treatments and active therapy. The discrepancies in results could be attributed to the limited number of studies, small sample sizes present in both of the aforementioned analyses (10, 48), and variations in inclusion criteria. Thomas et al. (10) reported limited or insignificant effects of acupuncture on reducing nocturnal enuresis. However, our study was constrained by the available research and thus unable to explore this aspect. Similarly, considering acupoint stimulation therapy for PSUI, Li et al. (49) suggested in their analysis that moxibustion considerably alleviates urinary incontinence symptoms and reduces the severity of such occurrences. However, prior systematic reviews have not comprehensively evaluated the impact of EA on bladder function in patients with PSUI, particularly using objective indicators such as MCC and RUV employed in our study.

### 4.3 The effect of acupuncture on PSUI

Our results indicated that in terms of responder rate, there were no significant differences between EA and active treatments, and the active interventions included in this study consisted of techniques such as catheterization, bladder function training, and medication therapy, which are widely utilized in clinical practice. Similar no significant differences occurred when comparing EA combined with basic treatments to basic treatments alone. Notably, substantial heterogeneity was observed within these comparisons. However, the addition of EA to both basic treatments and active treatments led to an improvement in responder rate, with low heterogeneity.

Concerning the 24-h urinary incontinence frequency, EA demonstrated a decrease in urinary incontinence occurrences compared to sham EA (as determined through narrative analysis). This positive effect was also observed to persist to the 3-month follow-up period. In addition, EA, as a supplementary treatment to both basic treatments and the combination of basic treatments with active interventions, has the potential to reduce the frequency of urinary incontinence incidents. Simultaneously, an analysis (50) revealed that EA was not able to improve the frequency of incontinence episodes in mixed urinary incontinence patients during the 72-h period. The ICIQ-SF is used for the assessment of urinary incontinence symptoms and demonstrates a strong correlation with the 24-h pad test (51). In terms of the ICIQ-SF, the addition of EA to both basic treatments and active treatments led to a decrease in ICIQ-SF scores, indicating a potential improvement in urinary function and symptom severity. Uroflowmetry is considered the “gold standard” for assessing lower urinary tract function as it provides a prompt, comprehensive, and objective evaluation of bladder storage and voiding capabilities (52). Regarding the MCC, descriptive analysis showed that EA was superior to sham EA and could persist for up to 3 months after treatment (26). Meta-analysis indicated that the combination of EA with basic treatments or basic treatments along with active treatments led to an improvement in MCC scores. Similarly, there was also an improvement in RUV outcomes. Remarkably, none of the included studies reported any instances of severe adverse reactions. EA-related adverse events were mild reactions such as pain and subcutaneous bleeding.

The analysis conducted by TSA, which relied on response rate and MCC outcomes, indicated that the sample size was sufficient to draw conclusions, implying that further clinical studies might not be needed. However, it is advisable to interpret these findings with caution as the level of certainty in the results ranged from moderate to low or very low. Hence, it is recommended to approach these results carefully and emphasize the need for additional high-quality clinical research.

Furthermore, it is worth noting that due to the uniqueness of acupuncture treatment, which emphasizes personalized approaches, and the variations in incorporating acupuncture treatment plans in many aspects, we did not conduct subgroup analyses for EA treatment regimens. However, in general, as previously mentioned, the acupoints used most frequently are primarily located in the lower abdomen and lumbosacral region. According to traditional Chinese medicine theory of “needling at the site of the disease,” electrical stimulation through acupuncture can contribute to the recovery of this area. Different stimulation parameters can have varying effects on acupoint stimulation, but which stimulation parameter is optimal requires further confirmation through clinical research.

### 4.4 Mechanism of electroacupuncture

The regulation of urination involves both bladder filling and emptying (53), and any damage to the central and peripheral nervous systems governing its function can lead to urinary abnormalities. Following a stroke, central regulatory mechanisms related to urination are impaired, leading to urinary incontinence due to factors such as detrusor muscle overactivity (54), impaired urinary awareness (55), and cognitive function decline (56). While EA has shown promising therapeutic effects in the treatment of post-stroke urinary incontinence, its underlying mechanisms remain incompletely understood. The current research study suggests that electroacupuncture may alleviate ischemic brain injury by inhibiting glycolysis and increasing the levels of certain neuroprotective metabolites (57). It has been observed to suppress the production of intravascular inflammatory factors, reduce the infarct area, and promote angiogenesis and glial cell proliferation for repair within the infarcted region (58, 59). Furthermore, it can activate functional reorganization in the peri-infarct area (60) and enhance bladder contraction function (61). Electroacupuncture downregulates the expression of the proto-oncogene c-fos within the pontine micturition center, thereby modulating the coordinated function between the detrusor and the urethral sphincter (62). Moreover, it can also be effective by enhancing post-stroke cognitive function (63).

### 4.5 Implications for future research and practice

As PSUI is a secondary condition of stroke, the treatment of the primary disease (stroke) is of importance. Whether in the experimental or control group, fundamental internal medical treatment should be considered as the primary choice, and its detailed implementation should be comprehensively documented within the research study.

Among the studies included in this research, only one study conducted a comparative assessment of the clinical efficacy between EA and sham EA for PSUI. Sham EA can be employed in future clinical studies, such as utilizing non-acupoint superficial needling in the application of EA. Some of the studies included in this review did not adhere to the CONSORT statement (64) and the Standards for Reporting Interventions in Clinical Trials of Acupuncture (STRICTA) (65, 66). It is recommended that for future clinical trials, there should be a more detailed categorization of recruited patients based on the type, severity, and duration of urinary incontinence. Rigorous adherence to the CONSORT statement and STRICTA guidelines for study design and reporting is essential. Accurate methods for calculating sample sizes, description of random sequence generation, allocation concealment, blinding procedures, and comprehensive reporting of adverse events throughout the study should be implemented. In the review, a considerable number of the included studies employed the responder rate as the primary outcome. It is recommended that urinary pad test assessment be used as an objective metric to provide a more reliable assessment of efficacy in the treatment of urinary incontinence. Additionally, follow-up is also a crucial component in future research endeavors.

### 4.6 Limitations

Our systematic review and meta-analysis included the latest RCTs and assessed the effect of EA on PSUI. However, this study does have certain limitations. While assessing publication bias for the primary outcome using Egger’s test, the results indicated the presence of publication bias in the included studies (*p* < 0.05). This bias may be attributed to the potential omission of unpublished literature during the inclusion process as well as the fact that all included studies in this review originated from China. The majority of the studies included in this review exhibited low quality, particularly with significant risks observed in the implementation of allocation concealment and blinding procedures; due to the unique nature of acupuncture therapy, research implementation is challenging. The majority of included studies originate from China, where acupuncture has its roots and is widely practiced. Blinding of participants and personnel is often limited, and there is a high risk of bias. Due to the constraints of the included studies, subgroup analysis based on urinary incontinence types could not be performed. Furthermore, the evidence from this study’s findings is predominantly of low quality.

## 5 Conclusion

EA as an adjunctive treatment can be effective for patients with PSUI. Furthermore, to enhance the strength and reliability of future research, it is advisable to conduct multicenter, large-sample clinical studies with minimal bias risks that can offer robust and dependable evidence.

## Data availability statement

The original contributions presented in the study are included in the article/Supplementary material, further inquiries can be directed to the corresponding author.

## Author contributions

ZJ: Conceptualization, Data curation, Formal analysis, Methodology, Software, Writing – original draft, Writing – review & editing. NZ: Conceptualization, Data curation, Methodology, Software, Writing – review & editing. GL: Data curation, Formal analysis, Writing – review & editing. XS: Data curation, Methodology, Writing – review & editing. XC: Methodology, Supervision, Writing – review & editing. DM: Investigation, Supervision, Writing – review & editing. MG: Investigation, Supervision, Writing – review & editing. SW: Investigation, Methodology, Writing – review & editing. HZ: Funding acquisition, Supervision, Writing – review & editing.

## Footnotes

^1^ https://www.crd.york.ac.uk/prospero/display_record.php?ID=CRD42023449599

## References

[1] Saini V Guada L Yavagal DR. Global epidemiology of stroke and access to acute ischemic stroke interventions. Neurology. (2021) 97:S6–S16. doi: 10.1212/WNL.0000000000012781, PMID: 34785599

[2] Owolabi MO Thrift AG Mahal A Ishida M Martins S Johnson WD . Primary stroke prevention worldwide: translating evidence into action. Lancet Public Health. (2022) 7:e74–85. doi: 10.1016/S2468-2667(21)00230-9, PMID: 34756176 PMC8727355

[3] Tu WJ Wang LD. China stroke surveillance report 2021. Mil Med Res. (2023) 10:33. doi: 10.1186/s40779-023-00463-x, PMID: 37468952 PMC10355019

[4] Jacob L Kostev K. Urinary and fecal incontinence in stroke survivors followed in general practice: a retrospective cohort study. Ann Phys Rehabil Med. (2020) 63:488–94. doi: 10.1016/j.rehab.2019.12.007, PMID: 31981836

[5] Fowler CJ . Neurological disorders of micturition and their treatment. Brain. (1999) 122:1213–31. doi: 10.1093/brain/122.7.1213 10388789

[6] Pettersen R Haig Y Nakstad PH Wyller TB. Subtypes of urinary incontinence after stroke: relation to size and location of cerebrovascular damage. Age Ageing. (2008) 37:324–7. doi: 10.1093/ageing/afm196, PMID: 18250094

[7] Pizzol D Demurtas J Celotto S Maggi S Smith L Angiolelli G . Urinary incontinence and quality of life: a systematic review and meta-analysis. Aging Clin Exp Res. (2021) 33:25–35. doi: 10.1007/s40520-020-01712-y, PMID: 32964401 PMC7897623

[8] Smith C Almallouhi E Feng W. Urinary tract infection after stroke: a narrative review. J Neurol Sci. (2019) 403:146–52. doi: 10.1016/j.jns.2019.06.005, PMID: 31288133

[9] Tuong NE Klausner AP Hampton LJ. A review of post-stroke urinary incontinence. Can J Urol. (2016) 23:8265–70. PMID: 27347618

[10] Thomas LH Coupe J Cross LD Tan AL Watkins CL. Interventions for treating urinary incontinence after stroke in adults. Cochrane Database Syst Rev. (2019) 2019:CD004462. doi: 10.1002/14651858.CD004462.pub4, PMID: 30706461 PMC6355973

[11] Pearlman A Kreder K. Evaluation and treatment of urinary incontinence in the aging male. Postgrad Med. (2020) 132:9–17. doi: 10.1080/00325481.2020.1831790, PMID: 33017202

[12] Maurer V Stahlberg J Schiffmann I Marks P Rosenbaum CM Soave A . Continence and complication rates of artificial urinary sphincter devices (ams 800) for parkinson and stroke patients with incontinence after prostate surgery: retrospective analysis of a prospective database. Urol Int. (2021) 105:225–31. doi: 10.1159/000512051, PMID: 33440398

[13] Mao JJ Liou KT Baser RE Bao T Panageas KS Romero S . Effectiveness of electroacupuncture or auricular acupuncture vs usual care for chronic musculoskeletal pain among cancer survivors: the peace randomized clinical trial. JAMA Oncol. (2021) 7:720–7. doi: 10.1001/jamaoncol.2021.0310, PMID: 33734288 PMC7974834

[14] Wang W Liu Y Yang X Sun J Yue Z Lu D . Effects of electroacupuncture for opioid-induced constipation in patients with cancer in China: a randomized clinical trial. JAMA Netw Open. (2023) 6:e230310. doi: 10.1001/jamanetworkopen.2023.0310, PMID: 36811861 PMC9947731

[15] Yin X Li W Liang T Lu B Yue H Li S . Effect of electroacupuncture on insomnia in patients with depression: a randomized clinical trial. JAMA Netw Open. (2022) 5:e2220563. doi: 10.1001/jamanetworkopen.2022.20563, PMID: 35797047 PMC9264041

[16] Tu JF Yang JW Shi GX Yu ZS Li JL Lin LL . Efficacy of intensive acupuncture versus sham acupuncture in knee osteoarthritis: a randomized controlled trial. Arthritis Rheumatol. (2021) 73:448–58. doi: 10.1002/art.41584, PMID: 33174383

[17] Liu Z Liu Y Xu H He L Chen Y Fu L . Effect of electroacupuncture on urinary leakage among women with stress urinary incontinence: a randomized clinical trial. JAMA. (2017) 317:2493–501. doi: 10.1001/jama.2017.7220, PMID: 28655016 PMC5815072

[18] Liu H Jiang Y Wang N Yan H Chen L Gao J . Scalp acupuncture enhances local brain regions functional activities and functional connections between cerebral hemispheres in acute ischemic stroke patients. Anat Rec. (2021) 304:2538–51. doi: 10.1002/ar.24746, PMID: 34431612 PMC9290874

[19] Cho SY Kim M Sun JJ Jahng GH Kim HJ Park SU . A comparison of brain activity between healthy subjects and stroke patients on fmri by acupuncture stimulation. Chin J Integr Med. (2013) 19:269–76. doi: 10.1007/s11655-013-1436-4, PMID: 23546630

[20] Wang Y Chen Y Meng L Wu B Ouyang L Peng R . Electro-acupuncture treatment inhibits the inflammatory response by regulating gammadelta t and treg cells in ischemic stroke. Exp Neurol. (2023) 362:114324. doi: 10.1016/j.expneurol.2023.114324, PMID: 36669751

[21] Xu H Wang Y Luo Y. Otulin is a new target of EA treatment in the alleviation of brain injury and glial cell activation via suppression of the nf-kappab signalling pathway in acute ischaemic stroke rats. Mol Med. (2021) 27:37. doi: 10.1186/s10020-021-00297-0, PMID: 33836646 PMC8035756

[22] Wang H Chen S Zhang Y Xu H Sun H. Electroacupuncture ameliorates neuronal injury by pink1/parkin-mediated mitophagy clearance in cerebral ischemia-reperfusion. Nitric Oxide. (2019) 91:23–34. doi: 10.1016/j.niox.2019.07.004, PMID: 31323277

[23] Cui Y Li Q Wang D Bao R Li L Zhu J . Does electroacupuncture benefit mixed urinary incontinence? A systematic review and meta-analysis with trial sequential analysis. Int Urogynecol J. (2022) 33:751–66. doi: 10.1007/s00192-021-05057-6, PMID: 35088093 PMC9021078

[24] Lai X Zhang J Chen J Lai C Huang C. Is electroacupuncture safe and effective for treatment of stress urinary incontinence in women? A systematic review and meta-analysis. J Int Med Res. (2020) 48:030006052094833. doi: 10.1177/0300060520948337, PMID: 33045874 PMC7570783

[25] Zhong Y Song Y Zeng F Zhao Y Black B Guan Y. Effectiveness of electroacupuncture for female stress urinary incontinence: a systematic review and meta-analysis. J Tradit Chin Med. (2020) 40:707–20. doi: 10.19852/j.cnki.jtcm.2020.05.001 33000572

[26] Liu Y Liu L Wang X. Electroacupuncture at points baliao and huiyang (bl35) for post-stroke detrusor overactivity. Neural Regen Res. (2013) 8:1663–72. doi: 10.4103/1673-5374.121661, PMID: 25206463 PMC4145909

[27] Jiang C Lu LN. Clinical observation on electroacupuncture combined with moxibustion for treatment of urinary incontinence after stroke due to deficiency of kidney-yang. Zhen Ci Yan Jiu. (2020) 45:578–82. doi: 10.13702/j.1000-0607.200300, PMID: 32705834

[28] Chu JM Bao YH Zou C Zhao HL Gong Y Wang CM. Randomized controlled clinical trials for electroacupuncture treatment of urinary incontinence in stroke patients. Zhen Ci Yan Jiu. (2011) 36:428–32. doi: 10.13702/j.1000-0607.2011.06.009 PMID: 22379789

[29] Song FJ Jiang SH Zheng SL Ye TS Zhang H Zhu WZ . Electroacupuncture for post-stroke urinary incontinence: a multi-center randomized controlled study. Zhongguo Zhen Jiu. (2013) 33:769–73. doi: 10.13703/j.0255-2930.2013.09.002 PMID: 24298760

[30] Higgins JPT Green S. (editors). Cochrane handbook for systematic reviews of interventions version 5.1.0. *[updated March 2011]. The Cochrane Collaboration*. (2011) Available at: https://www.handbook.cochrane.org.

[31] Thorlund K Devereaux PJ Wetterslev J Guyatt G Ioannidis JP Thabane L . Can trial sequential monitoring boundaries reduce spurious inferences from meta-analyses? Int J Epidemiol. (2009) 38:276–86. doi: 10.1093/ije/dyn179, PMID: 18824467

[32] Brok J Thorlund K Wetterslev J Gluud C. Apparently conclusive meta-analyses may be inconclusive--trial sequential analysis adjustment of random error risk due to repetitive testing of accumulating data in apparently conclusive neonatal meta-analyses. Int J Epidemiol. (2009) 38:287–98. doi: 10.1093/ije/dyn188 18824466

[33] Guyatt GH Oxman AD Schunemann HJ Tugwell P Knottnerus A. Grade guidelines: a new series of articles in the journal of clinical epidemiology. J Clin Epidemiol. (2011) 64:380–2. doi: 10.1016/j.jclinepi.2010.09.011, PMID: 21185693

[34] Nie X Li L Ren K Ren C Wang Z. Efficacy evaluation of electroacupuncture in treatment of urinary incontinence after cerebral hemorrhage. J Hubei Univ Tradition Chin Med. (2022) 24:89–91. doi: 10.1097/MD.0000000000022275

[35] Wang Q Yang Z Song Q Tang K Ming S Jiang F . Therapeutic evaluation of electroacupuncture on urge urinary incontinence patients after stroke. China J Tradition Chin Med Pharmacy. (2022) 37:6180–3.

[36] Liu L Li H Chen S Xu J. Effects of electroacupuncture at zhongji and guanyuan on urgency urinary incontinence after stroke. Chin J Rehabil Theory Pract. (2020) 26:93–7. doi: 10.3969/j.issn-1006.9771.2019.00.018

[37] Wu Y Yang Y. Effects of electroacupuncture at baliao points combined with rehabilitation training on neurogenic bladder dynamics after stroke. J Emerg Tradition Chin Med. (2019) 28:496–8. doi: 10.3969/j.issn.1004-745X.2019.03.033

[38] Wang G Song X Teng X. Clinical observation on electroacupuncture treatment of post-stroke urinary incontinence. J Clin Acupunct Moxibust. (2017) 33:22–4. doi: 10.3969/j.issn.1005-0779.2017.06.007

[39] Zhao J Hong L. Clinical observation of electro-acupuncture combined with pelvic floor muscle training in treatment of patients with urinary incontinence after cerebral infarction. China J Tradition Chin Med Pharmacy. (2016) 31:3377–80.

[40] Song F Jiang S Zheng S Ye T Zhang H Zhu W . Electroacupuncture treatment for post-stroke urinary incontinence: a multi-center randomized controlled study. Chin Acupunct Moxibust. (2013) 33:769–73., PMID: 24298760

[41] Zhang Q Cui Q Song B. Clinical observation of electroacupuncture combined with pelvic floor muscle exercise in the treatment of post-stroke urinary incontinence in 50 cases. Chin J Tradition Med Sci Technol. (2012) 19:83–4. doi: 10.3969/j.issn.1005-7072.2012.01.063

[42] Chu J Bao Y Zou C Zhao H Gong Y Wang C. Clinical observation of electroacupuncture treatment for non-inhibitory neurogenic bladder. Chin Arch Tradit Chin Med. (2012) 30:2293–5. doi: 10.13193/j.archtcm.2012.10.151.chujm.026

[43] Zhou G Wang M Tao H Wang Q Jin J. Clinical efficacy observation of electroacupuncture treatment in 46 patients with post-stroke urinary incontinence. Chin J Phys Med Rehabil. (2012) 34:462–4. doi: 10.3760/cma.j.issn.0254-1424.2012.06.016

[44] Chen J. Efficacy and safety evaluation of electroacupuncture treatment for urgency urinary incontinence after stroke. Beijing: Beijing University of Chinese Medicine (Master’s thesis) (2008).

[45] He Y. Clinical study of acupuncture and moxibustion in the treatment of post-stroke urinary incontinence. Guangzhou: Guangzhou University of Traditional Chinese Medicine (Master’s thesis) (2008).

[46] Liu Z Du Y. Evaluation of the curative effect of electroacupunture on post apoplectic incontinence of urine. Shanghai J Acupunct Moxibust. (2007) 10:13–4. doi: 10.13460/j.issn.1005-0957.2007.10.007

[47] Liu L. Study on the treatment of acute stroke-related urinary disorders by electroacupuncture, urodynamic and related factors. Jilin: Jilin University (Doctoral dissertation (2006).

[48] Thomas LH Cross S Barrett J French B Leathley M Sutton CJ . Treatment of urinary incontinence after stroke in adults. Cochrane Database Syst Rev. (2008) 2008:CD004462. doi: 10.1002/14651858.CD004462.pub3, PMID: 18254050 PMC6464794

[49] Li X Li ZM Tan JY Wang T Chen JX Chen X . Moxibustion for post-stroke urinary incontinence in adults: a systematic review and meta-analysis of randomized controlled trials. Complement Ther Clin Pract. (2021) 42:101294. doi: 10.1016/j.ctcp.2020.101294, PMID: 33360387

[50] Long Z Chen H Yu S Wang X Liu Z. Effect of acupuncture for mixed urinary incontinence in women: a systematic review. Front Public Health. (2022) 10:827853. doi: 10.3389/fpubh.2022.827853, PMID: 35372235 PMC8971660

[51] Karantanis E Fynes M Moore KH Stanton SL. Comparison of the iciq-sf and 24-hour pad test with other measures for evaluating the severity of urodynamic stress incontinence. Int Urogynecol J Pelvic Floor Dysfunct. (2004) 15:111–6; discussion 116. doi: 10.1007/s00192-004-1123-2, PMID: 15014938

[52] Zhang HT Nong QP Wei L Liang XN Liang YP Jia YH . Effect of thunder-fire moxibustion combined with electroacupuncture on urodynamics in patients with neurogenic bladder after spinal cord injury. Zhen Ci Yan Jiu. (2021) 46:958–62. doi: 10.13702/j.1000-0607.20210690, PMID: 34865334

[53] Fowler CJ Griffiths D de Groat WC. The neural control of micturition. Nat Rev Neurosci. (2008) 9:453–66. doi: 10.1038/nrn2401, PMID: 18490916 PMC2897743

[54] Liu Y Xu G Luo M Teng HF. Effects of transcutaneous electrical nerve stimulation at two frequencies on urinary incontinence in poststroke patients: a randomized controlled trial. Am J Phys Med Rehabil. (2016) 95:183–93. doi: 10.1097/PHM.0000000000000360 26259053

[55] Pettersen R Stien R Wyller TB. Post-stroke urinary incontinence with impaired awareness of the need to void: clinical and urodynamic features. BJU Int. (2007) 99:1073–7. doi: 10.1111/j.1464-410X.2007.06754.x, PMID: 17437440

[56] Kushner DS Johnson-Greene D. Association of urinary incontinence with cognition, transfers and discharge destination in acute stroke inpatient rehabilitation. J Stroke Cerebrovasc Dis. (2018) 27:2677–82. doi: 10.1016/j.jstrokecerebrovasdis.2018.05.028, PMID: 29941393

[57] Yang SH Zhang XX Zhong ZQ Luo XX Wang YF Xiao XP . Metabolomics analysis of electroacupuncture pretreatment induced neuroprotection on mice with ischemic stroke. Am J Chin Med. (2023) 51:1127–51. doi: 10.1142/S0192415X23500520, PMID: 37335209

[58] Wang L Sheng G Cui J Yao Y Bai X Chen F . Electroacupuncture attenuates ischemic injury after stroke and promotes angiogenesis via activation of epo mediated src and vegf signaling pathways. PLoS One. (2022) 17:e274620. doi: 10.1371/journal.pone.0274620, PMID: 36108080 PMC9477374

[59] Shih CC Hsu YT Wang HH Chen TL Tsai CC Lane HL . Decreased risk of stroke in patients with traumatic brain injury receiving acupuncture treatment: a population-based retrospective cohort study. PLoS One. (2014) 9:e89208. doi: 10.1371/journal.pone.0089208 24586597 PMC3929662

[60] Du Y Shi L Li J Xiong J Li B Fan X. Angiogenesis and improved cerebral blood flow in the ischemic boundary area were detected after electroacupuncture treatment to rats with ischemic stroke. Neurol Res. (2011) 33:101–7. doi: 10.1179/016164110X12714125204317, PMID: 20546685

[61] Liu Z Wang W Wu J Zhou K Liu B. Electroacupuncture improves bladder and bowel function in patients with traumatic spinal cord injury: results from a prospective observational study. Evid Based Complement Alternat Med. (2013) 2013:543174. doi: 10.1155/2013/543174, PMID: 24382977 PMC3870613

[62] Chung IM Kim YS Sung YH Kim SE Ko IG Shin MS . Effects of acupuncture on abdominal leak point pressure and c-fos expression in the brain of rats with stress urinary incontinence. Neurosci Lett. (2008) 439:18–23. doi: 10.1016/j.neulet.2008.04.100, PMID: 18502581

[63] Zhang Y Mao X Lin R Li Z Lin J. Electroacupuncture ameliorates cognitive impairment through inhibition of ca (2+)-mediated neurotoxicity in a rat model of cerebral ischaemia-reperfusion injury. Acupunct Med. (2018) 36:401–7. doi: 10.1136/acupmed-2016-011353, PMID: 30257960 PMC6287559

[64] Moher D Schulz KF Altman D. The consort statement: revised recommendations for improving the quality of reports of parallel-group randomized trials. JAMA. (2001) 285:1987–91. doi: 10.1001/jama.285.15.1987, PMID: 11308435

[65] MacPherson H White A Cummings M Jobst KA Rose K Niemtzow RC. Standards for reporting interventions in controlled trials of acupuncture: the stricta recommendations. J Altern Complement Med. (2002) 8:85–9. doi: 10.1089/107555302753507212 11890439

[66] MacPherson H Altman DG Hammerschlag R Youping L Taixiang W White A . Revised standards for reporting interventions in clinical trials of acupuncture (stricta): extending the consort statement. J Evid Based Med. (2010) 3:140–55. doi: 10.1111/j.1756-5391.2010.01086.x, PMID: 21349059

