# Supplementary material for: Electroacupuncture for post-stroke urinary incontinence: a systematic review and meta-analysis with trial sequential analysis
Source: Front Neurol. 2023 Nov 15;14:1282580. doi: 10.3389/fneur.2023.1282580 (PMC10693342; doi:10.3389/fneur.2023.1282580)
Supplement: Supplementary file 1 [file Data_Sheet_1.docx]

**Chinese database:**

**CNKI:**

(主题=针灸 + 针刺 + 电针) AND (主题=中风 + 脑出血 + 脑梗死 + 脑血管意外 + 脑卒中) AND (主题=尿失禁 + 小便失禁 + 排尿障碍) AND (全文=随机)

Results: 294

**Wanfang:**

主题:(针灸 OR 针刺 OR 电针) and 主题:(中风 OR 脑出血 OR 脑梗死 OR 脑血管意外 OR 脑卒中) and 主题:(尿失禁 OR 小便失禁 OR 排尿障碍)

Results: 462

**VIP:**

((((((题名或关键词=针灸 OR 题名或关键词=针刺) OR 题名或关键词=电针) AND ((((题名或关键词=中风 OR 题名或关键词=脑出血) OR 题名或关键词=脑梗死) OR 题名或关键词=脑血管意外) OR 题名或关键词=脑卒中))) AND ((题名或关键词=尿失禁 OR 题名或关键词=小便失禁) OR 题名或关键词=排尿障碍)))

Results: 219

**CBM:**

( "针灸"[常用字段:智能] OR "针刺"[常用字段:智能] OR "电针"[常用字段:智能]) AND( "中风"[常用字段:智能] OR "脑出血"[常用字段:智能] OR "脑梗死"[常用字段:智能] OR "脑血管意外"[常用字段:智能] OR "脑卒中"[常用字段:智能]) AND( "尿失禁"[常用字段:智能] OR "小便失禁"[常用字段:智能] OR "排尿障碍"[常用字段:智能])

Results: 575

**English database:**

**PubMed:**

#1 “Stroke”[MeSH Terms]

#2 “Cerebrovascular accident”[Title/Abstract] OR “CVA”[Title/Abstract] OR “Brain vascular accident”[Title/Abstract] OR “Cerebral Infarction”[Title/Abstract] OR “Apoplexy”[Title/Abstract]

#3 #1 OR #2

#4 “urinary incontinence”[MeSH Terms] OR “Urinary Incontinence, Urge”[MeSH Terms] OR “Urinary Incontinence, Stress”[MeSH Terms] OR “Diurnal Enuresis”[MeSH Terms]

#5 “effort incontinen*”[Title/Abstract] OR “involuntary urination”[Title/Abstract] OR “leaking of urine”[Title/Abstract] OR “leakage of urine”[Title/Abstract] OR “urinary leak*”[Title/Abstract] OR “urine leak*”[Title/Abstract]

#6 #4 OR #5

#7 “electroacupuncture”[MeSH Terms] OR “Acupuncture Therapy”[MeSH Terms]

#8 “Acupuncture”[MeSH Terms] OR “Electric acupuncture”[MeSH Terms] OR “Electrical acupuncture”[MeSH Terms]

#9 #7 OR #8

#10 “Randomized Controlled Trials as Topic”[MeSH Terms] OR “random allocation”[MeSH Terms]

#11 “randomized”[Title/Abstract] OR “randomly”[Title/Abstract] OR “RCT”[Title/Abstract] OR “trial”[Title/Abstract] OR “Randomized Controlled Trial”[Publication Type] OR “clinical trial”[Publication Type]

#12 #10 OR #11

#13 #3 AND #6 AND #9 AND #12

Results:12

**Web of Science:**

#1: (((((((((TS=(urinary incontinence)) OR TS=(Urinary Incontinence, Urge)) OR TS=(Urinary Incontinence, Stress)) OR TS=(Diurnal Enuresis)) OR TS=(effort incontinen*)) OR TS=(involuntary urination)) OR TS=(leaking of urine)) OR TS=(leakage of urine)) OR TS=(urinary leak*)) OR TS=(urine leak*)

#2: (((((TS=(Apoplexy)) OR TS=(Cerebral Infarction)) OR TS=(Brain vascular accident)) OR TS=(CVA)) OR TS=(Cerebrovascular accident)) OR TS=(Stroke)

#3: ((((TS=(electroacupuncture)) OR TS=(Acupuncture Therapy)) OR TS=(Acupuncture)) OR TS=(Electric acupuncture)) OR TS=(Electrical acupuncture)

#4: (((((AB=(random allocation)) OR AB=(randomized)) OR AB=(randomly)) OR AB=(RCT)) OR AB=(Randomized Controlled Trial*)) OR AB=(trial*)

#1 AND #2 AND #3 AND #4

Results:12

**Embase:**

#1: 'stroke patient'/exp OR 'cerebrovascular accident'/exp OR stroke:ab,ti OR cva:ab,ti OR 'brain vascular accident':ab,ti OR 'cerebral infarction':ab,ti OR apoplexy:ab,ti

#2:'acupuncture'/exp OR 'electroacupuncture'/exp OR 'electric acupuncture':ab,ti OR 'electrical acupuncture':ab,ti

#3:'urinary incontinence, urge':ti OR 'urinary incontinence, stress':ti OR 'diurnal enuresis':ti OR 'effort incontinence':ti OR 'involuntary urination':ti OR 'leaking of urine':ti OR 'leakage of urine':ti OR 'urinary leak*':ti OR 'urine leak*':ti OR 'urine incontinence'/exp OR 'stress incontinence'/exp OR 'urge incontinence'/exp

#4:'randomized controlled trial'/exp OR 'trail'/exp OR 'clinical study'/exp OR 'clinical'/exp OR 'random allocation':ab,ti OR randomized:ab,ti OR randomly:ab,ti OR rct:ab,ti OR trial:ab,ti

#1 AND #2 AND #3 AND #4

Results:28

**Cochrane Library:**

ID Search Hits

#1 MeSH descriptor: [Acupuncture] explode all trees 713

#2 (electroacupuncture OR acupuncture):ti,ab,kw (Word variations have been searched) 19866

#3 MeSH descriptor: [Stroke] explode all trees 14924

#4 (Cerebrovascular accident OR CVA OR Brain vascular accident OR Cerebral Infarction OR Apoplexy):ti,ab,kw (Word variations have been searched) 23773

#5 MeSH descriptor: [Urinary Incontinence] explode all trees 3096

#6 ((Cerebrovascular accident) OR CVA OR (Brain vascular accident) OR (Cerebral Infarction) OR ApoplexyDiurnal Enuresis OR effort incontinence OR involuntary urination OR leaking of urine OR leakage of urine OR urinary leak* OR urine leak*):ti,ab,kw (Word variations have been searched) 25391

#7 MeSH descriptor: [Randomized Controlled Trial] explode all trees 25732

#8 #1 OR #2 19866

#9 #3 OR #4 33466

#10 #5 OR #6 27970

#11 #8 AND #9 AND #10 AND #7 26

Results: 26
